# Micronutrient status in nursing home residents: associations with dietary supplementation and health characteristics in the cross-sectional multicentre Nutricare study

**DOI:** 10.1093/ageing/afaf290

**Published:** 2025-10-09

**Authors:** Živa Lavriša, Hristo Hristov, Neža Hren, Sanja Krušič, Nadan Gregorič, Igor Pravst

**Affiliations:** University of Ljubljana, Biotechnical faculty, Ljubljana, Slovenia; Institute of Nutrition, Ljubljana, Slovenia; Institute of Nutrition, Ljubljana, Slovenia; National Institute of Public Health, Ljubljana, Slovenia; Institute of Nutrition, Ljubljana, Slovenia; Institute of Nutrition, Ljubljana, Slovenia; University Medical Centre Ljubljana, Ljubljana, Slovenia; University of Ljubljana, Medical Faculty, Ljubljana, Slovenia; University of Ljubljana, Biotechnical faculty, Ljubljana, Slovenia; Institute of Nutrition, Ljubljana, Slovenia; VIST - Faculty of Applied Sciences, Ljubljana, Slovenia

**Keywords:** Micronutrient deficiency, nursing home residents, vitamin D, micronutrient intake, anaemia, older people

## Abstract

Micronutrient deficiencies are common in institutionalised older adults due to chronic diseases, functional decline and age-related changes, increasing their risk of poor health outcomes. The study aim was to assess the usual daily intakes and status of selected micronutrients in nursing home (NH) residents, and to examine the relationship between dietary supplementation, micronutrient status and health characteristics. Additionally, we aimed to identify factors associated with serum 25-hydroxy-vitamin D (25(OH)D) and haemoglobin levels. The Nutricare study included 387 NH residents with low to moderate care needs from 20 NH. Collected data included resident characteristics, usual dietary intake (two 24-hour recalls, food frequency questionnaire), blood/serum biomarkers (25(OH)D, folate, vitamin B12, haemoglobin, ferritin), hand grip strength and body composition, estimated by bioelectrical impedance spectroscopy. Over 90% of study participants had inadequate intakes of vitamin D, magnesium and potassium. Despite 63% of participants reported use of supplements, suboptimal micronutrient status was common, with those aged 80+ and with higher care needs being more at risk. Most of participants who did not supplement vitamin D had insufficient 25(OH)D levels—both during winter (98%) and summer (71%). Low haemoglobin (<130 g/L) was found in 43% and ferritin <30 μg/L in 9%. These findings highlight the need for optimised NH menus and personalised supplementation strategies. Year-round vitamin D supplementation is essential, as diet and sun exposure are insufficient. Priority should be given to prevention over treatment, supported by clear guidelines for micronutrient management in this vulnerable population.

## Key Points

- Micronutrient deficiencies are widespread in nursing home residents, particularly for vitamin D, magnesium and potassium.
- 63% reported supplement use but suboptimal micronutrient status remained common.
- Most non-supplementers had insufficient vitamin D levels year-round, highlighting the inadequacy of diet and sun exposure alone.
- Low haemoglobin and ferritin levels were frequent, with implications for anaemia and overall health in older adults.
- These findings underscore the urgent need for improved NH menus, individualised supplementation, and preventive micronutrient strategies guided by clear protocols.

## Introduction

Nursing home (NH) residents are particularly vulnerable to nutritional inadequacies due to ageing-related changes, chronic conditions and institutional dietary constraints [1]. Limited dietary diversity and altered eating abilities further increase the risk of micronutrient deficiencies, which are linked to poor health outcomes, functional decline and frailty [2–4]. Beyond inadequate intake, chronic diseases and medications can impair micronutrient absorption and metabolism, compounding deficiency risks [5].

In NH residents, inadequate intake and deficiency of vitamin D, vitamin B12, folate and iron is frequently observed, despite the availability of supplementation [3]. Vitamin D is especially critical for musculoskeletal health and fall prevention [6, 7]. Yet, although highly prevalent [8], deficiency often goes unrecognised and untreated in this setting [9], even though it is cost-effective and safe [10, 11]. Anaemia, also widespread amongst NH residents, is commonly associated with nutrient deficiencies and can significantly impair quality of life [12]. It is commonly defined using WHO haemoglobin thresholds of <130 g/L for men and <120 g/L for women [13], which remain standard in both clinical and research settings. Although haemoglobin levels tend to decline with age, recent large-scale data suggest that these changes may reflect at least partly physiological processes. However, the appropriateness of age-specific haemoglobin reference limits remains controversial due to the difficulty in defining a truly healthy older population [14].

Despite clear evidence on the benefits and safety of supplementation, micronutrient management remains under prioritised in long-term care. In NH settings, the high prevalence of polypharmacy and chronic diseases requires careful management of micronutrient status, as certain conditions and medications can increase nutrient needs or hinder absorption [15]. However, clear guidelines on who and when should receive supplementation, seem to be often lacking, leaving decisions largely to individual physicians, like it was exposed in the Danish case of vitamin D and calcium supplementation [16]. Additionally, oversight of supplementation may be limited, as the availability of supplements on the market can hinder proper monitoring. While adequate dietary intake is key to preventing deficiencies, it is not always sufficient. NH menus should therefore be carefully planned to ensure nutritional adequacy, and when needed, appropriate and controlled oral nutritional supplementation can help prevent deficiencies, support functional status, and reduce healthcare costs [17].

Despite existing national and international dietary surveys, comprehensive data on the micronutrient intake and status of institutionalised older adults remain limited. Most studies rely on general population data, which do not capture the unique nutritional risks and care-related constraints present in NH. To explore these challenges, the Nutricare project was established in Slovenia to investigate dietary challenges in the population of NH residents.

The objective of this study was to assess the usual daily intakes and status of selected micronutrients in NH residents, and to examine the relationship between supplementation, micronutrient status and health characteristics. Additionally, we aimed to identify factors associated with serum 25-hydroxyvitamin D and haemoglobin levels in this population group.

This study addresses that critical gap by providing detailed, national multicentre data on dietary intake, supplementation and blood biomarkers in a large sample of Slovenian NH residents. The multicentre design enhances representativeness across diverse regions and institutional practices, offering robust insight into national-level trends and informing tailored nutritional strategies and public health guidelines for this vulnerable population.

## Methods

### Study design

This cross-sectional study, part of the national Nutricare project, assessed the nutrition of NH residents across all Slovenian health regions. Data were collected in both summer and winter 2022/2023 to account for seasonal variation, involving 387 residents with low to moderate care needs and no severe cognitive impairment, according to NH medical records [18].

### Data collection and variables

Age, sex, care category, medical history, chronic diseases and prescribed medications were obtained from NH and medical records. Sociodemographic factors, eating habits and nonprescription drug use were assessed via a questionnaire. Physical activity, anthropometrics, hand grip strength and body composition (bioimpedance) were measured. Dietary intake was evaluated using 24-hour dietary recalls (24HDR) and a food frequency questionnaire (FFQ), while micronutrient status was determined through analysis of blood biomarkers.

### Hand grip strength, physical activity, anthropometry and body composition

Weight and height were measured using a Seca 799 medical scale (Seca GmbH, Hamburg, Germany). Hand grip strength, classified as low below 16 kg for females and 27 kg for males [19] was assessed with a Jamar dynamometer (J.A. Preston Corporation, Clifton, NJ). Body composition was estimated using bioelectrical impedance spectroscopy with a Bodystat Multiscan 5000 (Bodystat, Isle of Man, Ireland); fat mass (FM) and fat-free mass were calculated via Deurenberg *et al.*’s equation [20], and appendicular skeletal muscle mass using Sergi *et al.*’s equation [21] (cut-offs: <20 kg for males, <15 kg for females) [19]. Participants were categorised to ‘low’, ‘moderate’ and ‘high’ activity level according to International Physical Activity Questionnaire (IPAQ) [22]. Timed Up and Go test was used to assess functional ability of participants [23] with cut-offs below and above 20 seconds [19]. Detailed description of all performed measurements can be found elsewhere [24].

### Dietary intakes and the use of supplements

Data on dietary intakes was collected by two 24HDR, which took place at least 7 days apart and combined with FFQ data to determine usual daily dietary intakes. The intake of micronutrients was analysed using Open Platform for Clinical Nutrition [25]. Mis-reporters were handled by the procedure described by Black *et al.* [26]. Detailed description of the procedure was previously published [18]. Supplementation of micronutrients was recorded from medical records (medically prescribed) and by questionnaire (nonmedically prescribed supplements). Exact supplements were recorded, including the brand, active ingredients and the amount used. Daily amounts were calculated for each participant and supplement.

### Analysis of blood biomarkers

Fasting blood samples of the participants was collected. Samples were transported to the accredited medical diagnostic laboratory (Adrialab/Synlab, Ljubljana, Slovenia) for analysis. The analyses of vitamin B12 [27], haemoglobin and ferritin [28], folate [29], C-reactive protein [24] and 25-hydroxy-vitamin D (25(OH)D) [30] were previously described. Transformations of values for specific blood biomarkers that were below the limit of detection (LOD) were transformed to a constant value by the formula: LOD/$\sqrt{2}$. Values above the LOD, were substituted with the LOD. Cut-offs used for serum biomarkers were as follows. Vitamin D: < 30 nmol/L—deficiency, <50 nmol/L—insufficiency, >75 nmol/L—optimal [31]; vitamin B12: <148 pmol/L—very low, <221 pmol/L—low, >221 pmol/L—optimal [32]; folate: <7 nmol/L—very low, <10 nmol/L—low [33]; haemoglobin: <120 and < 130 g/L—low [13]; ferritin: <15 μg/L [34]. For haemoglobin, we applied these established thresholds to ensure comparability with other studies and to align with current clinical practise in the absence of universally accepted age-specific criteria.

### Data analysis

Usual micronutrient intakes were estimated using the Multiple Source Method (MSM) [35], a statistical approach that accounts for within-subject variation and was described previously [18]. This method combines repeated 24HDRs with FFQ data to estimate usual intake distributions at the individual level. It adjusts for within-person day-to-day variation and between-person differences, while the inclusion of FFQ data improves estimates of long-term dietary patterns by compensating for the limited recall days. All models for the estimation of micronutrient intakes were adjusted including age, sex and daily energy intake as covariates. Results were presented as means with standard deviations (SD), medians and percentiles (5%, 50% and 95%), stratified by sex and age group.

Micronutrient intakes were assessed against national dietary reference values (DRV) [36], while micronutrient status was evaluated based on means and the proportion of participants falling within specific deficiency cut-offs related to disease risk and anthropometric characteristics.

Factors associated with vitamin D and haemoglobin deficiency were examined using multivariate ordinal logistic regression analysis, applying two risk cut-offs for each outcome: 30 and 50 nmol/L for vitamin D deficiency and insufficiency, respectively, and 120 and 130 g/L for haemoglobin deficiency.

All statistical analyses were conducted using STATA (version 17.0; StataCorp LLC, College Station, TX, USA), with MSM applied to estimate usual dietary intake distributions.

## Results

**Figure 1:**
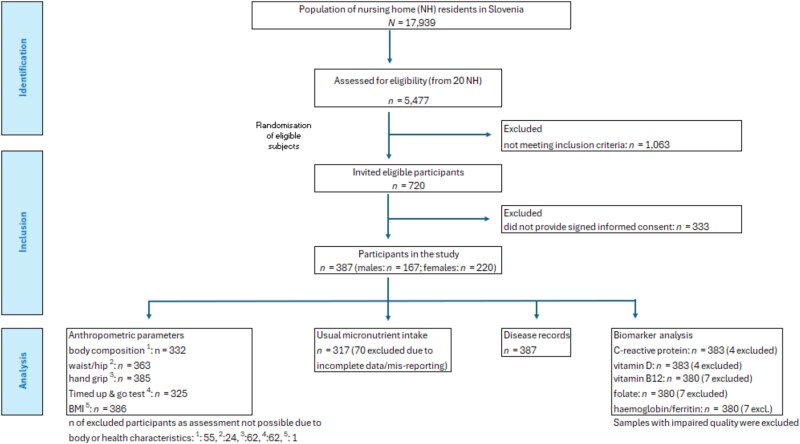
Participant flow chart.

In the Nutricare study, 387 participants, aged 65–101 years were included. We were unable to perform all measurements in all participants; Figure 1 shows the recruitment and sample sizes. The characteristics of the NH residents included in the study are presented in the Table 1. In the Nutricare study, most participants (96%) were from care categories with lower care requirements, therefore the results are applicable to these care groups. Altogether, 58.7% of participants were aged 80 years or older with females making a slight majority (56.8%).

**Table 1:** Usual daily micronutrient intakes and proportion (%) of the study population meeting DRV.

**Micronutrients**	**Female** (*n* = 181)					**Male** (*n* = 136)				
	Mean (SD)	Median	P5 (P95)	*n* (%) below DRV	Dietary reference values (DRV)	Mean (SD)	Median	P5 (P95)	*n* (%) below DRV	Dietary reference values (DRV)
Magnesium (mg)	215.8 (38.9)	213.27	158.6 (275.4)	178 (98.3)	300	235.6 (46.8)	224.87	159 (317.2)	133 (97.8)	350
Iron (mg)	9.3 (2.2)	9.18	6 (13.4)	109 (60.2)	10	10.4 (2.2)	10.45	6.4 (14.2)	54 (39.7)	10
Folate (μg)	278.2 (69.5)	272.96	171.8 (405.1)	122 (67.4)	300	308.6 (73.4)	306.07	191.4 (433.8)	61 (44.9)	300
Vitamin B12 (μg)	2.9 (1.2)	2.63	1.5 (5.2)	160 (88.4)	4.0	3 (1.2)	2.85	1.7 (5.2)	112 (82.4)	4
Vitamin D (μg)	3 (2.5)	2.30	1.1 (8.3)	181 (100)	20	3.3 (2.8)	2.50	1.4 (13.2)	136 (100)	20
Zinc (mg)	8.4 (2)	8.64	5.4 (11.6)	48 (26.5)	7.0	9.4 (1.8)	9.53	6.4 (12.5)	90 (66.2)	10
Calcium (mg)	904.2 (207.6)	888.77	602.2 (1272.9)	125 (69.1)	1000	959.5 (239.3)	948.75	569.6 (1323.2)	82 (60.3)	1000
Vitamin A (mg)	0.7 (0.3)	0.65	0.3 (1.2)	129 (71.3)	0.8	0.7 (0.3)	0.66	0.2 (1.2)	126 (92.6)	1.0
Vitamin B6 (mg)	1.9 (0.5)	1.89	1.3 (2.5)	18 (9.9)	1.4	2.2 (0.8)	2.04	1.5 (3.5)	13 (9.6)	1.6
Riboflavin (mg)	1.7 (0.4)	1.67	7 (19.2)	2 (1.1)	1.0	1.9 (0.6)	1.80	7.2 (24.5)	11 (8.1)	1.3
Vitamin E (mg)	12.7 (4.3)	12.04	1.1 (2.4)	77 (42.5)	11	15.3 (5.2)	14.91	1.2 (3.1)	33 (24.3)	12
Potassium (mg)	2864.3 (464.5)	2844.28	2155.9 (3669.0)	179 (98.9)	4000	3070.9 (508.1)	3054.38	2311.7 (4005.3)	129 (94.9)	4000
Selenium (μg)	62.4 (32.2)	54.09	28.2 (128.7)	108 (59.7)	60	67.6 (31.8)	60.90	33.3 (157)	95 (69.9)	70
Vitamin C (mg)	127.7 (56.8)	118.71	68.5 (263.7)	53 (29.3)	95	134.6 (74.9)	124.15	71.9 (202)	45 (33.1)	110
Thiamine (mg)	1.6 (0.6)	1.49	0.9 (3.1)	16 (8.8)	1.0	1.8 (0.7)	1.70	1 (3.4)	13 (9.6)	1.1
Niacin (mg)	35.5 (8.3)	35.19	22.4 (48.6)	0 (0)	1.1	38.9 (10.1)	38.70	23.5 (56.4)	0 (0)	1.4
Vitamin K (μg)	151.6 (55.9)	144.07	72.5 (251)	4 (2.2)	65	158.3 (53.4)	149.77	85.7 (269.9)	3 (2.2)	80
Phosphorus (mg)	1308.8 (228.2)	1295.90	931.5 (1676.8)	0 (0)	700	1428.8 (268.3)	1435.56	1020.9 (1837.2)	0 (0)	700
Vitamin B5 (mg)	5 (1.3)	4.87	3.4 (7.1)	154 (85.1)	6.0	5.9 (2.4)	5.32	3.7 (9.9)	89 (65.4)	6.0

Note: SD, standard deviation; P5, 5th percentile; P95, 95th percentile.

### Usual micronutrient intakes in nursing home residents

Regardless of sex, no participants met DRV for intake of vitamin D and at least 95% of participants did not meet DRV for magnesium and potassium intake (Table 1). On the other hand, usual dietary intakes (UDI) for phosphorus were approximately twice the DRV in all participants, which is consistent with international data. In general, males and females above 80 years of age had higher prevalence of not meeting DRV than those below 80 years (Supplementary tables 1 and 2).

### Micronutrient status in relation to participants’ characteristics and presence of chronic disease

Overall, 63% of participants used at least one micronutrient supplement, most commonly vitamin D (57%), with higher use amongst females (67%) than males (42%) (Table 2). Supplementers had higher mean serum 25(OH)D levels (70.0 vs. 31.3 nmol/L), though 56.5% still had suboptimal levels. Pill users had significantly higher 25(OH)D concentrations (79.1 vs. 67.4 nmol/L for drops), and supplementation was most common in those with osteoporosis (89.6%) (Supplementary table 3). Statistical modelling amongst individuals not supplementing with 25(OH)D (Supplementary Table 4) showed increased odds of lower 25(OH)D levels in those with low IPAQ scores compared with participants with medium (OR = 2.53, *P* < .01) and high (OR = 6.20, *P* = .01) activity levels. Lower 25(OH)D status was also associated with the winter season (OR = 2.72, *P* < .01), and a notable association was observed with an increasing number of chronic diseases (OR = 1.28, *P* = .07).

**Table 2:** Serum 25-hydroxyvitamin D (25(OH)D) status in all, vitamin D supplementing and non-supplementing participants and its association with participants’ characteristics and presence of diseases.

Variables	All *n* (%)	Suppl. *n* (%)	Serum 25(OH)D (nmol/L)—all subjects					Serum 25(OH)D (nmol/L)—vitamin D supplementers					Serum 25(OH)D (nmol/L)—vitamin D non-supplementers				
			Mean (SD)	Median	*n* (%)			Mean (SD)	Median	*n* (%)			Mean (SD)	Median	*n* (%)		
					< 30	< 50	< 75			< 30	< 50	< 75			< 30	< 50	< 75
Total	383 (100)	217 (56.7)	53.1 (31.5)	51.4	115 (30.0)	186 (48.6)	284 (74.2)	70.0 (27.9)	70.6	19 (8.8)	46 (21.3)	122 (56.5)	31.3 (20.6)	23.5	96 (57.5)	140 (83.8)	162 (97.0)
Sex																	
Female	220 (56.8)	147 (66.8)	56.3 (31.2)	55.5	59 (27.1)	95 (43.6)	153 (70.2)	69.4 (27.0)	69.3	13 (8.9)	34 (23.3)	83 (56.8)	29.6 (20.4)	22.7	46 (63.9)	61 (84.7)	70 (97.2)
Male	167 (43.2)	70 (41.9)	48.9 (31.4)	47.7	56 (33.9)	91 (55.2)	131 (79.4)	71.1 (29.9)	71.5	6 (8.6)	12 (17.1)	39 (55.7)	32.6 (20.7)	24.0	50 (52.6)	79 (83.2)	92 (96.8)
Age cohorts																	
<80 years	160 (41.3)	77 (48.1)	51.5 (31.7)	50.0	52 (32.9)	79 (50.0)	119 (75.3)	72.8 (26)	71.6	6 (7.8)	12 (15.6)	41 (53.2)	31.3 (21.9)	22.5	46 (56.8)	67 (82.7)	78 (96.3)
>80 years	227 (58.7)	140 (61.7)	54.2 (31.3)	52.7	63 (28.0)	107 (47.6)	165 (73.3)	68.4 (28.9)	67.9	13 (9.4)	34 (24.5)	81 (58.3)	31.3 (19.3)	23.8	50 (58.1)	73 (84.9)	84 (97.7)
Care category																	
1	272 (70.3)	156 (57.4)	54.1 (31.8)	51.2	75 (27.9)	132 (49.1)	196 (72.9)	70.1 (28.7)	68.9	12 (7.7)	36 (23.2)	86 (55.5)	32.4 (21)	25.0	63 (55.3)	96 (84.2)	110 (96.5)
2	100 (25.8)	51 (51.0)	49.5 (31.0)	49.2	36 (36.4)	50 (50.5)	77 (77.8)	69.2 (26.6)	71.6	6 (11.8)	9 (17.6)	30 (58.8)	28.6 (19.5)	22.6	30 (62.5)	41 (85.4)	47 (97.9)
3	15 (3.9)	10 (66.7)	58.7 (29.7)	60.2	4 (26.7)	4 (26.7)	11 (73.3)	71.2 (24.7)	70.0	1 (10)	1 (10.0)	6 (60.0)	33.5 (22.9)	23.5	3 (60)	3 (60.0)	5 (100)
IPAQ score																	
Low	139 (35.9)	72 (51.8)	48.3 (30.9)	49.7	54 (39.4)	69 (50.4)	104 (75.9)	70.2 (23.4)	72.3	6 (8.3)	10 (13.9)	39 (54.2)	24.0 (16.6)	19.0	48 (73.8)	59 (90.8)	65 (100)
Moderate	230 (59.4)	136 (59.1)	55.2 (31.5)	51.5	58 (25.4)	110 (48.2)	168 (73.7)	69.9 (29.9)	68.4	12 (8.9)	34 (25.2)	78 (57.8)	33.8 (19.4)	30.7	46 (49.5)	76 (81.7)	90 (96.8)
High	18 (4.7)	9 (50.0)	63.5 (31.9)	64.9	3 (16.7)	7 (38.9)	12 (66.7)	68.7 (34.2)	68.9	1 (11.1)	2 (22.2)	5 (55.6)	58.3 (30.5)	49.2	2 (22.2)	5 (55.6)	7 (77.8)
Smoking																	
Yes	54 (14.0)	25 (46.3)	53.2 (34.1)	52.0	17 (32.7)	24 (46.2)	39 (75.0)	73.4 (30.0)	71.4	3 (12.0)	5 (20.0)	13 (52.0)	34.5 (26.4)	20.2	14 (51.9)	19 (70.4)	26 (96.3)
No	333 (86.0)	192 (57.7)	53.1 (31.1)	51.2	98 (29.6)	162 (48.9)	245 (74.0)	69.5 (27.7)	69.9	16 (8.4)	41 (21.5)	109 (57.1)	30.7 (19.3)	23.6	82 (58.6)	121 (86.4)	136 (97.1)
Season																	
Winter	190 (49.1)	108 (56.8)	50.8 (31.8)	46.7	60 (31.9)	104 (55.3)	144 (76.6)	69.4 (28.8)	68.1	7 (6.5)	25 (23.4)	63 (58.9)	26.3 (14.1)	22.5	53 (65.4)	79 (97.5)	82 (100)
Summer	197 (50.9)	109 (55.3)	55.3 (31.1)	55.9	55 (28.2)	82 (42.1)	140 (71.8)	70.5 (27.2)	71.6	12 (11.0)	21 (19.3)	59 (54.1)	36.0 (24.3)	28.2	44 (50.0)	61 (70.9)	82 (94.2)
No chronic disease present	64 (17.6)	30 (46.9)	47.6 (28.1)	46.7	24 (35.3)	38 (55.9)	56 (82.4)	69.1 (24.1)	71.5	3 (10.0)	5 (16.7)	18 (60.0)	30.6 (17.6)	24.3	21 (55.3)	33 (86.8)	38 (100)
Chronic disease present	319 (82.4)	187 (58.6)	54.3 (32.1)	53.2	91 (28.9)	148 (47.0)	228 (72.4)	70.1 (28.6)	69.3	16 (8.6)	41 (22.0)	104 (55.9)	31.5 (21.4)	23.2	75 (58.1)	107 (82.9)	124 (96.1)
Sarcopenia	91 (27.6)	50 (54.9)	52.5 (29.8)	51.3	26 (28.9)	43 (47.8)	69 (76.7)	68.2 (26.9)	68.3	4 (8)	11 (22.0)	30 (60.0)	32.8 (20)	24.3	22 (55)	32 (80.0)	39 (97.5)
Diabetes mellitus type 2	89 (23.0)	44 (49.4)	51.5 (34.4)	46.8	34 (38.6)	49 (55.7)	63 (71.6)	75.4 (29.1)	78.1	3 (6.8)	10 (22.7)	21 (47.7)	27.5 (19.2)	20.1	31 (70.5)	39 (88.6)	42 (95.5)
Kidney disease	55 (14.2)	33 (60.0)	55.3 (29.8)	50.5	13 (24.1)	27 (50.0)	39 (72.2)	69.3 (24.9)	71.6	1 (3.0)	8 (24.2)	20 (60.6)	33.3 (23)	23.7	12 (57.1)	19 (90.5)	19 (90.5)
Osteoporosis	48 (12.4)	43 (89.6)	66.4 (24.5)	67.9	4 (8.5)	11 (23.4)	31 (66.0)	67.7 (24.4)	67.9	3 (7.0)	10 (23.3)	27 (62.8)	52.7 (24.4)	60.0	1 (25)	1 (25)	4 (100)
Heart failure	41 (10.6)	22 (53.7)	59.1 (37.9)	51.8	12 (30.0)	19 (47.5)	24 (60.0)	80.4 (32.9)	82.2	2 (9.1)	4 (18.2)	8 (36.4)	33.1 (25.5)	24.6	10 (55.6)	15 (83.3)	16 (88.9)
Thyroid disease	33 (8.5)	24 (72.7)	57.5 (27.9)	59.8	7 (21.9)	11 (34.4)	22 (68.8)	69.2 (21.2)	67.9	1 (4.2)	3 (12.5)	14 (58.3)	22.5 (10.3)	22.1	6 (75)	8 (100)	8 (100)
Hypertension	249 (64.3)	155 (62.2)	55.9 (33.0)	53.7	68 (27.8)	110 (44.9)	172 (70.2)	70.9 (29.7)	69.8	14 (9.1)	33 (21.4)	85 (55.2)	30.6 (20.5)	22.5	54 (59.3)	77 (84.6)	87 (95.6)
Hypercholesterolemia	69 (17.8)	43 (62.3)	54.9 (32.4)	53.3	21 (30.9)	30 (44.1)	44 (64.7)	71.6 (24.7)	77.6	2 (4.7)	8 (18.6)	21 (48.8)	26.3 (22.6)	18.5	19 (76)	22 (88)	23 (92)

Note: SD, standard deviation; IPAQ, International Physical Activity; Suppl., Supplementing.

About 10% of participants supplemented vitamin B12, more often females and those in higher care category, though status was similar across groups. Folate deficiency (<7 nmol/L) was seen in 2.3% of supplementers vs. 25.6% of non-supplementers, with suboptimal folate most common in the highest care category (93.3%) (Table 3).

**Table 3:** Serum concentrations of vitamin B12, folate, haemoglobin and ferritin in relation to participants’ characteristics and presence of diseases, regardless of supplementation.

Variables	Vitamin B12 (pmol/L)				Folate (nmol/L)				Haemoglobin (g/L)				Ferritin (μg/L)		
	Suppl. (%)	Mean (SD)	<148 (%)	<221 (%)	Suppl. (%))	Mean (SD)	<7 (%)	<10 (%)	Suppl. (%)	Mean (SD)	<120 (%)	<130 (%)	Mean (SD)	<30 (%)	<50 (%)
Total (*n* = 380)		283.6 (332.4)	22.1	55.5		14.7 (14.4)	22.9	47.4		131.4 (15.7)	23.4	43.4	174.5 (222.2)	10.8	22.7
Supplementing															
Yes	9.7	275.1 (279.1)	21.4	54.0	11.6	40.4 (8.0)	2.3	9.1	4.2	131.8 (15.8)	31.3	75.0	167.5 (184.0)	0.8	4.5
No	90.3	294.6 (391.7)	23.0	57.6	88.4	11.3 (23.8)	25.6	52.4	95.8	122.8 (11.3)	21.1	42.0	174.8 (224.0)	11.6	22.9
Sex															
Female	10.2	288.0 (321.5)	20.4	51.4	12.5	15.3 (15.2)	19.9	43.1	5.1	127.7 (14.3)	31.2	51.2	133.1 (140.7)	15.3	29.4
Male	9.1	277.8 (347.1)	24.4	61.0	10.4	14.0 (13.4)	26.8	53.0	3.0	136.3 (16.3)	13.3	33.3	228.9 (288.9)	6.7	14.5
Age cohorts															
<80 years	8.9	296.3 (368.5)	20.4	55.4	14.0	15.3 (15.2)	26.1	41.1	2.5	135.1 (15.7)	16.6	34.4	193.0 (268.7)	10.8	19.6
>80 years	10.3	274.6 (304.9)	23.3	55.6	9.9	14.3 (13.8)	20.6	47.5	9.8	128.9 (15.3)	28.3	49.8	161.4 (182.1)	12.1	25.3
Care category															
1	9.7	268.2 (277.0)	21.3	56.2	10.5	15.0 (13.8)	18.0	42.3	3.7	133.1 (15.2)	21.0	38.6	171.3 (216.6)	12.0	20.8
2	8.2	297.1 (302.5)	23.5	56.1	14.3	14.9 (16.7)	31.6	54.1	3.1	129.8 (15.0)	23.5	50.0	159.6 (187.2)	10.2	29.3
3	20.0	468.9 (925.1)	26.7	40.0	13.3	7.3 (2.1)	53.3	93.3	20.0	112.9 (18.7)	66.7	86.7	327.7 (419.1)	13.3	20.0
IPAQ score															
Low	10.2	301.4 (405.6)	24.1	59.9	13.9	15.1 (15.9)	27.7	54.0	4.4	130.5 (16.4)	26.3	43.1	151.4 (232.0)	10.9	23.4
Moderate	9.8	276.0 (292.4)	21.3	53.3	10.7	14.6 (13.9)	21.3	43.6	4.4	131.9 (15.5)	22.7	45.3	188.6 (221.7)	11.6	23.2
High	5.6	243.0 (122.3)	16.7	50.0	5.6	13.0 (6.4)	5.6	44.4	0	132.7 (14.3)	11.1	22.2	173.8 (125.8)	16.7	16.7
Smoking															
Yes	11.5	266.2 (284.7)	25.0	59.6	15.4	14.8 (16.3)	34.6	55.8	3.8	137.9 (16.5)	13.5	25	184.9 (201.7)	13.5	22.7
No	9.5	286.3 (339.6)	21.6	54.9	11.0	14.7 (14.1)	21.0	46.0	4.3	130.4 (15.4)	25.0	46.3	172.8 (225.5)	11.3	25.0
No chronic disease present	6.0	333.1 (497.9)	19.4	52.2	13.4	15.7 (17.3)	25.4	50.7	3.0	134.1 (13.8)	22.4	34.3	177.3 (168.9)	9.0	16.2
Chronic disease present	8.3	273.0 (284.8)	22.6	55.0	11.1	14.5 (13.7)	22.2	46.3	4.5	130.9 (16.1)	23.6	45.4	173.9 (232.3)	12.1	24.4
Sarcopenia	6.7	264.8 (203.1)	21.3	52.2	6.7	12.1 (10.5)	31.1	51.1	5.6	127.8 (16.4)	34.4	52.2	229.4 (338.4)	12.4	28.9
Diabetes mellitus type 2	8.0	286.4 (280.1)	25.3	55.2	8.0	12.6 (8.7)	18.2	43.2	4.7	130.0 (17.5)	28.7	48.3	148.8 (183.2)	11.5	23.9
Kidney disease	9.3	287.1 (407.6)	25.9	55.6	25.9	20.6 (21.9)	24.1	38.9	9.3	126.4 (17.1)	37.0	53.7	174.8 (233.6)	13.0	25.9
Osteoporosis	8.5	351.1 (471.4)	14.9	46.8	12.8	16.0 (18.5)	23.4	48.9	0	125.9 (14.7)	40.4	57.4	127.0 (102.7)	6.4	27.7
Heart failure	7.5	266.7 (278.4)	15.0	60.0	10.0	15.4 (17.2)	30.0	50.0	7.5	129.5 (15.4)	25.0	47.5	211.0 (229.8)	15.0	22.5
Thyroid disease	12.5	237.5 (199.3)	25.0	65.6	15.6	12.8 (11.6)	28.1	59.4	9.4	125.7 (12.5)	34.4	65.6	129.5 (146.1)	18.8	34.4
Hypertension	8.2	277.4 (295.3)	22.0	53.9	11.8	14.8 (14.4)	19.6	46.9	4.1	130.3 (15.8)	24.3	46.5	164.6 (219.3)	12.3	26.1
Hypercholesterolemia	8.8	266.7 (190.6)	19.1	48.5	8.8	16.3 (17.3)	20.6	38.2.	1.5	133.1 (14.2)	19.1	38.2	189.8 (311.8)	11.8	22.1

Note: IPAQ, International Physical Activity score; Suppl., Supplementing.

Altogether, 43.4% of participants had haemoglobin concentrations <130 g/L (23.4% < 120 g/L). Participants older than 80 years (OR = 1.92, *P* < .01) and those in higher care categories (OR = 2.17, *P* < .001) were at greater risk of low haemoglobin. In addition, higher C- reactive protein (CRP) values (OR = 1.03, *P* = .019), female sex (OR = 2.13, *P* < .001), and low-FM (OR = 2.29, *P* = .031) were associated with increased odds of lower haemoglobin levels amongst participants not supplementing with iron (Supplementary Table 4). Results also indicated greater variability and overall lower haemoglobin concentrations in the iron-supplementing group, suggesting that other factors may hinder the maintenance of adequate haemoglobin levels. Furthermore, females were more vulnerable to low ferritin concentrations (<30 μg/L) compared with males, and risk was also elevated amongst participants older than 80 years, those in higher care categories, smokers, and individuals with chronic diseases (Table 3).

Multivariate ordinal logistic regression (Supplementary table 4) identified some statistically significant associations with vitamin D and haemoglobin status but overall added limited explanatory value. Seasonal variation and physical activity were linked to lower odds of vitamin D deficiency, while hand grip strength, male sex and FM % showed some association with haemoglobin levels. However, effect sizes were modest, and the findings offered little additional insight beyond known relationships.

## Discussion

This study aimed to evaluate the usual intake and status of selected micronutrients in NH residents and explore the relationship between micronutrient status, chronic diseases and supplementation practices. Although mean UDI were below reference values for 9/19 micronutrients, notably few participants met recommendations for vitamin D, potassium and magnesium—nutrients essential for frailty prevention [37], chronic disease management [38], musculoskeletal function [39] and quality of life in geriatric individuals. Higher potassium and lower sodium intake are linked to lower cardio-vascular disease (CVD) risk and better hypertension control [40], which affects over 60% of participants in our sample. Excessive salt intake remains common in Slovenia [41], further increasing cardiovascular risk. Similar micronutrient inadequacies were observed in other studies [3]. To improve micronutrient status in NH residents, menu optimization is key. Increasing fruits, vegetables, wholegrains and legumes, currently underrepresented in Slovenian NH menus [18], and reducing sodium-rich foods like processed meats could enhance both micronutrient and fibre intake. Fortified foods may also be beneficial in NH settings, where reduced appetite and intake are common [42].

Although 63% participants were supplementing their diets, which is considerably more than the 14% reported in a Dutch study [43], many exhibited suboptimal micronutrient status despite the supplementation. As foods are a limited source of vitamin D, supplementation is widely recommended for NH residents, yet remains poorly implemented [8, 16, 44]. Our findings are in line with results of studies in other countries [45], showing that vitamin D is often prescribed for osteoporosis [46]. Although supplementation improved mean 25(OH)D levels in these residents, nearly half remained below optimal levels, suggesting insufficient dosage, malabsorption or poor compliance. Notably, differences were observed between pharmaceutical forms: unsupervised use of liquid drops may be less practical for older adults than tablets or capsules, potentially leading to lower adherence. However, data on compliance of vitamin D supplementation were not available in our study, which limits our ability to assess adherence-related factors contributing to low serum 25(OH)D levels. Vitamin D status was presented descriptively by season to reflect the epidemiologic situation rather than to test seasonal differences statistically. Considerable variability was observed within each season, likely reflecting multiple factors beyond sunlight exposure in NH residents. As mean and median concentrations were insufficient in both winter and summer, our findings indicate that supplementation is warranted year-round. Given broader health benefits of vitamin D, year-round supplementation for all NH residents is a safe, cost-effective strategy that prioritises prevention over treatment of deficiency [8, 16, 47].

Our previous study showed concerning vitamin B12 status in Slovenian NH residents, particularly amongst the oldest and male participants [48]. Despite its clinical relevance, approximately half of the participants with chronic conditions where vitamin B12 supplementation could be beneficial, had suboptimal vitamin B12 status, and supplementation was rare. Additionally, targeted folate supplementation may be warranted in residents with greater care needs.

Optimal micronutrient status is key to preventing frailty, as deficiencies contribute to its progression [49]. Anaemia was far more prevalent in NH residents, than in community-dwelling older adults [28], despite sufficient iron intake, particularly in participants at higher frailty risk—those with weak grip strength, low body fat, and in females. Besides anaemia, suboptimal vitamin D and folate status may further increase frailty [50].

Our findings indicate that suboptimal micronutrient status can persist even with supplementation, underscoring the need for more targeted strategies. Establishing an optimal regimen is especially difficult in residents with multiple comorbidities. We also highlight that poor compliance may limit effectiveness if supplementation is not integrated into routine care. These results support a shift from deficiency treatment to proactive prevention.

A key strength of this study is the inclusion of NH residents from various regions of Slovenia, ensuring a representative sample, and in robust methodology of dietary assessment. Dietary intake was assessed using the MSM method, integrating two 24-hour recalls and the FFQ, to capture long-term dietary habits and minimise misreporting bias, while nutritional status was established using well-established blood biomarkers. Some study limitations also need to be mentioned. Study was focused on residents from lower care categories, excluding those with higher dependency. As a result, findings may not be fully generalizable to more dependent or cognitively impaired residents, who often present with distinct clinical and nutritional profiles, including greater prevalence of dysphagia, cognitive decline and feeding assistance needs. These factors may influence both dietary intake and biomarker status and thus warrant separate investigation. We should also mention that while vitamin B12 and folate status are sometimes assessed with additional consideration of serum homocysteine (Hcy) levels, in older population Hcy is commonly elevated and of very limited interpretative use [29], and was therefore not measured in our study. Furthermore, although ferritin levels were reported, they were not directly interpreted due to the known limitations of ferritin as an acute-phase reactant. Given the high prevalence of elevated CRP levels in this population, the potential for inflammation-driven elevation of ferritin values was recognised, and CRP levels were considered in the descriptive analysis to acknowledge this confounding effect. However, the presence of chronic low-grade inflammation may still limit the utility of ferritin as a reliable marker of iron status in this context.

The findings highlight key issues in achieving and maintaining adequate micronutrient status, warranting further research. Future studies should explore optimal supplementation strategies for individuals with multiple comorbidities, as well as the reasons for taking or not taking supplements. Additionally, cost analyses are needed to compare the expenses of preventive supplementation with the higher costs of treating deficiency-related complications, such as fractures or hospitalisations.

## Conclusion

This study highlights the widespread issue of suboptimal micronutrient intake and status in NH residents, underscoring the need for improved dietary and supplementation strategies. Enhancing menus with micronutrient-dense foods and regularly reviewing recipes could improve intake and support residents’ health. Given the high prevalence of vitamin D deficiency, routine supplementation throughout the year should be considered. For other micronutrients, a more individualised approach is needed, especially for the oldest, frailest and most disabled residents. Our findings show that suboptimal status can persist despite supplementation, indicating a need for routine screening and integration of supplementation into daily medication protocols. Staff training on identifying nutritional risks and appropriate use of supplements could further support implementation. Shifting from deficiency treatment to prevention and establishing clear, consensus-based guidelines for supplementation in older adults with comorbidities would enable more effective micronutrient status management.

## Supplementary Material

Supplementary_table_1_afaf290

Supplementary_table_2_afaf290

Supplementary_table_3_afaf290

Supplementary_table_4_afaf290

## Research data transparency and availability:

The raw data supporting the conclusions of this article can be made available by the authors by request.
