# Supplementary material for: Micronutrient status in nursing home residents: associations with dietary supplementation and health characteristics in the cross-sectional multicentre Nutricare study
Source: Age Ageing. 2025 Oct 9;54(10):afaf290. doi: 10.1093/ageing/afaf290 (PMC12510403; doi:10.1093/ageing/afaf290)
Supplement: Supplementary_table_1_afaf290 [file supplementary_table_1_afaf290.docx]

**Micronutrient status in nursing home residents: associations with dietary supplementation and health characteristics in the cross-sectional multicentre Nutricare study**

Supplementary table 1: Usual micronutrient intakes and proportion (%) of female study population, aged below and above 80 years, compared to dietary reference values

| **Micronutrients** | **Female** | | | | | | | | |
| --- | --- | --- | --- | --- | --- | --- | --- | --- | --- |
|  | < 80 years (*n* = 56) | | | | > 80 years (*n* = 125) | | | | Dietary reference values (DRV) |
|  | Mean (SD) | Median | P5 (P95) | *n* (%) below DRV | Mean (SD) | Median | P5 (P95) | *n* (%) below DRV |  |
| Magnesium (mg) | 223.6 (40.7) | 224.5 | 160.6 (285.6) | 54 (96.4) | 212.4 (37.8) | 212.6 | 155 (269.1) | 124 (99.2) | 300 |
| Iron (mg) | 9.6 (2.1) | 9.4 | 6.4 (12.8) | 31 (55.4) | 9.2 (2.3) | 9.1 | 5.5 (13.5) | 78 (62.4) | 10 |
| Folate (µg) | 286.3 (68.6) | 279.8 | 183.5 (392.2) | 37 (66.1) | 274.6 (69.8) | 260.2 | 171.1 (410) | 85 (68) | 300 |
| Vitamin B12 (µg) | 2.7 (1.1) | 2.3 | 1.5 (5.2) | 50 (89.3) | 2.9 (1.2) | 2.7 | 1.5 (5.2) | 110 (88) | 4 |
| Vitamin D (µg) | 2.6 (1.4) | 2.1 | 1.4 (5.8) | 56 (100) | 3.2 (2.8) | 2.3 | 1.1 (9.9) | 125 (100) | 20 |
| Zinc (mg) | 8.7 (2.1) | 8.9 | 5.3 (12.2) | 14 (25) | 8.3 (1.9) | 8.4 | 5.5 (11.3) | 34 (27.2) | 7 |
| Calcium (mg) | 910.8 (230) | 884.6 | 584.1 (1,314) | 37 (66.1) | 901.3 (197.6) | 888.8 | 605.6 (1,261.6) | 88 (70.4) | 1,000 |
| Vitamin A (mg) | 0.7 (0.2) | 0.7 | 0.3 (1.1) | 43 (76.8) | 0.7 (0.3) | 0.7 | 0.3 (1.3) | 86 (68.8) | 0.8 |
| Vitamin B6 (mg) | 2 (0.5) | 1.9 | 1.3 (2.8) | 3 (5.4) | 1.9 (0.4) | 1.9 | 1.2 (2.3) | 15 (12) | 1.4 |
| Riboflavin (mg) | 13.6 (3.8) | 13.9 | 8.2 (20.5) | 0 (0) | 12.3 (4.4) | 11.6 | 6.7 (18.7) | 2 (1.6) | 1 |
| Vitamin E (mg) | 1.8 (0.4) | 1.7 | 1.1 (2.5) | 19 (33.9) | 1.7 (0.4) | 1.7 | 1.1 (2.2) | 58 (46.4) | 11 |
| Potassium (mg) | 2,869.6 (509.1) | 2,860.8 | 1,939 (3,690.5) | 54 (96.4) | 2,861.9 (445.2) | 2,835.9 | 2,155.9 (3669) | 125 (100) | 4,000 |
| Selene (µg) | 59.2 (23.6) | 53.3 | 29.1 (114.1) | 34 (60.7) | 63.8 (35.3) | 55.3 | 26.4 (150.1) | 74 (59.2) | 60 |
| Vitamin C (mg) | 116.3 (32.5) | 118.9 | 72 (172.9) | 17 (30.4) | 132.8 (64.3) | 117.9 | 64.4 (288.8) | 36 (28.8) | 95 |
| Thiamine (mg) | 1.7 (0.6) | 1.7 | 1 (3.2) | 3 (5.4) | 1.6 (0.6) | 1.5 | 0.9 (3.1) | 13 (10.4) | 1 |
| Niacin (mg) | 37.1 (9.2) | 37.1 | 20.3 (52.5) | 0 (0) | 34.8 (7.8) | 34.3 | 22.4 (47.3) | 0 (0) | 1.1 |
| Vitamin K (µg) | 156.2 (50) | 147.5 | 89.1 (251) | 1 (1.8) | 149.5 (58.4) | 142.2 | 71 (259.8) | 3 (2.4) | 65 |
| Phosphorus (mg) | 1,358.7 (244.4) | 1,298.6 | 972.4 (1,738) | 0 (0) | 1,286.4 (217.8) | 1,295.9 | 930.9 (1,632.9) | 0 (0) | 700 |
| Vitamin B5 (mg) | 5.4 (1.4) | 5.1 | 3.4 (8.9) | 43 (76.8) | 4.8 (1.2) | 4.8 | 3.4 (6.5) | 111 (88.8) | 6 |
