# Supplementary material for: Micronutrient status in nursing home residents: associations with dietary supplementation and health characteristics in the cross-sectional multicentre Nutricare study
Source: Age Ageing. 2025 Oct 9;54(10):afaf290. doi: 10.1093/ageing/afaf290 (PMC12510403; doi:10.1093/ageing/afaf290)
Supplement: Supplementary_table_2_afaf290 [file supplementary_table_2_afaf290.docx]

**Micronutrient status in nursing home residents: associations with dietary supplementation and health characteristics in the cross-sectional multicentre Nutricare study**

Supplementary table 2: Usual micronutrient intakes and proportion (%) of the male study population, aged below and above 80 years, compared to dietary reference values

| **Micronutrients** | **Male** | | | | | | | | |
| --- | --- | --- | --- | --- | --- | --- | --- | --- | --- |
|  | < 80 years (*n* = 69) | | | | > 80 years (*n* = 67) | | | | Dietary reference values (DRV) |
|  | Mean (SD) | Median | P5 (P95) | N (%) below DRV | Mean (SD) | Median | P5 (P95) | N (%) below DRV |  |
| Magnesium (mg) | 238.6 (46.6) | 224.6 | 170.4 (317.8) | 67 (97.1) | 232.5 (47.1) | 226.4 | 158.5 (307) | 66 (98.5) | 350 |
| Iron (mg) | 10.5 (2.1) | 10.8 | 6.7 (14.2) | 23 (33.3) | 10.3 (2.4) | 10.2 | 6.4 (14.2) | 31 (46.3) | 10 |
| Folate (µg) | 315 (72.9) | 315.4 | 216.9 (447.8) | 29 (42) | 301.9 (73.8) | 300.8 | 185.4 (428.6) | 32 (47.8) | 300 |
| Vitamin B12 (µg) | 3.1 (1.2) | 3.0 | 1.6 (5.2) | 54 (78.3) | 2.8 (1.1) | 2.6 | 1.8 (4.7) | 58 (86.6) | 4 |
| Vitamin D (µg) | 3.7 (3.1) | 2.7 | 1.7 (13.4) | 69 (100) | 2.9 (2.3) | 2.4 | 1.3 (4.4) | 67 (100) | 20 |
| Zinc (mg) | 9.5 (1.8) | 9.7 | 6 (12.1) | 43 (62.3) | 9.3 (1.7) | 9.3 | 6.6 (12.6) | 47 (70.1) | 10 |
| Calcium (mg) | 981.5 (255.6) | 957.4 | 575.2 (1,342.3) | 41 (59.4) | 936.8 (221) | 932.7 | 553.3 (1,291.8) | 41 (61.2) | 1,000 |
| Vitamin A (mg) | 0.7 (0.4) | 0.7 | 0.2 (1) | 66 (95.7) | 0.7 (0.2) | 0.6 | 0.3 (1.2) | 60 (89.6) | 1 |
| Vitamin B6 (mg) | 2.2 (0.8) | 2.1 | 1.5 (4) | 6 (8.7) | 2.1 (0.7) | 2.0 | 1.5 (2.5) | 7 (10.4) | 1.6 |
| Riboflavin (mg) | 16 (5.3) | 15.2 | 9.5 (25.1) | 4 (5.8) | 14.5 (4.9) | 14.2 | 7.1 (20.6) | 7 (10.4) | 1.3 |
| Vitamin E (mg) | 2 (0.7) | 1.9 | 1.3 (3.1) | 13 (18.8) | 1.8 (0.6) | 1.7 | 1.2 (2.6) | 20 (29.9) | 12 |
| Potassium (mg) | 3,054.9 (530.2) | 3,025.4 | 2,267.2 (4,068.9) | 65 (94.2) | 3,087.3 (487.8) | 3,056.2 | 2,358.8 (3,999.1) | 64 (95.5) | 4,000 |
| Selenium (µg) | 73.5 (34) | 64.7 | 43.2 (168.4) | 42 (60.9) | 61.5 (28.3) | 56.1 | 31.8 (90.6) | 53 (79.1) | 70 |
| Vitamin C (mg) | 136.5 (94.7) | 124.4 | 66.5 (200.7) | 24 (34.8) | 132.7 (47.2) | 123.0 | 74.6 (202) | 21 (31.3) | 110 |
| Thiamine (mg) | 1.8 (0.7) | 1.7 | 1.1 (3.6) | 6 (8.7) | 1.8 (0.6) | 1.7 | 1 (3.4) | 7 (10.4) | 1.1 |
| Niacin (mg) | 39.8 (11.1) | 39.6 | 23.8 (58) | 0 (0) | 38 (9.1) | 38.2 | 23.5 (52.2) | 0 (0) | 1.4 |
| Vitamin K (µg) | 159 (50.7) | 151.7 | 88.8 (252.7) | 2 (2.9) | 157.7 (56.4) | 143.2 | 85 (285) | 1 (1.5) | 80 |
| Phosphorus (mg) | 1,467.3 (289.6) | 1,462.3 | 1,012.8 (1,891.9) | 0 (0) | 1,389.1 (240.1) | 1,389.0 | 1,034.1 (1,808.4) | 0 (0) | 700 |
| Vitamin B5 (mg) | 6.2 (2.7) | 5.7 | 3.8 (12) | 41 (59.4) | 5.5 (1.9) | 5.2 | 3.7 (7.2) | 48 (71.6) | 6 |
