# Supplementary material for: Micronutrient status in nursing home residents: associations with dietary supplementation and health characteristics in the cross-sectional multicentre Nutricare study
Source: Age Ageing. 2025 Oct 9;54(10):afaf290. doi: 10.1093/ageing/afaf290 (PMC12510403; doi:10.1093/ageing/afaf290)
Supplement: Supplementary_table_3_afaf290 [file supplementary_table_3_afaf290.docx]

**Micronutrient status in nursing home residents: associations with dietary supplementation and health characteristics in the cross-sectional multicentre Nutricare study**

Supplementary table 3: Serum 25(OH)D (nmol/L) concentration regarding different participants’ characteristics and season

| Variables | *n* | 25(OH)D (nmol/L) | | | | | | |
| --- | --- | --- | --- | --- | --- | --- | --- | --- |
|  |  | Mean (SD) | Median | P05 | P95 | <30 (%) | <50 (%) | <75 (%) |
| All (%) | 383 | 53.1 (31.5) | 51.4 | 10.7 | 103.1 | 30.0 | 48.6 | 74.2 |
| Sex |  |  |  |  |  |  |  |  |
| Female | 218 | 56.3 (31.2) | 55.5 | 12.0 | 110.0 | 27.1 | 43.6 | 70.2 |
| Male | 165 | 48.9 (31.4) | 47.7 | 10.2 | 101.3 | 33.9 | 55.2 | 79.4 |
| Age cohorts |  |  |  |  |  |  |  |  |
| <80 years | 158 | 51.5 (31.7) | 50.4 | 10.0 | 108.0 | 32.9 | 50.0 | 75.3 |
| >80 years | 225 | 54.2 (31.3) | 52.7 | 13.2 | 101.1 | 28.0 | 47.6 | 73.3 |
| Care category |  |  |  |  |  |  |  |  |
| 1 | 269 | 54.1 (31.8) | 51.2 | 11.0 | 108.1 | 27.9 | 49.1 | 72.9 |
| 2 | 99 | 49.5 (31.0) | 49.2 | 8.2 | 102.1 | 36.4 | 50.5 | 77.8 |
| 3 | 15 | 58.7 (29.7) | 60.2 | 11.5 | 112.1 | 26.7 | 26.7 | 73.3 |
| TUG |  |  |  |  |  |  |  |  |
| <20 seconds | 243 | 55.0 (31.2) | 53.2 | 11.5 | 103.1 | 27.2 | 46.5 | 72.8 |
| >20 seconds | 69 | 56.4 (34.9) | 53.2 | 10.2 | 112.1 | 27.5 | 46.4 | 68.1 |
| Hand grip |  |  |  |  |  |  |  |  |
| Low | 166 | 50.8 (29.3) | 48.9 | 10.2 | 98.6 | 30.7 | 51.8 | 77.7 |
| Normal | 213 | 54.4 (33.1) | 52.7 | 10.7 | 115.1 | 30.0 | 46.9 | 71.8 |
| Fat mass % |  |  |  |  |  |  |  |  |
| Low | 46 | 53.5 (35.4) | 55.0 | 9.5 | 119.6 | 34.8 | 47.8 | 69.6 |
| High | 283 | 53.9 (30.9) | 51.4 | 12.0 | 102.3 | 27.9 | 47.7 | 74.9 |
| IPAQ score |  |  |  |  |  |  |  |  |
| Low | 137 | 48.3 (30.9) | 49.7 | 8.7 | 99.6 | 39.4 | 50.4 | 75.9 |
| Moderate | 228 | 55.2 (31.5) | 51.5 | 14.0 | 106.8 | 25.4 | 48.2 | 73.7 |
| High | 18 | 63.5 (31.9) | 64.9 | 11.5 | 130.8 | 16.7 | 38.9 | 66.7 |
| Smoking |  |  |  |  |  |  |  |  |
| Yes | 52 | 53.2 (34.1) | 51.2 | 11.0 | 102.3 | 32.7 | 46.2 | 75.0 |
| No | 331 | 53.1 (31.1) | 52.0 | 9.5 | 116.8 | 29.6 | 48.9 | 75.0 |
| Season |  |  |  |  |  |  |  |  |
| Winter | 178 | 50.8 (31.8) | 46.7 | 11.0 | 102.0 | 31.9 | 55.3 | 76.6 |
| Summer | 195 | 55.3 (31.1) | 55.9 | 10.7 | 106.8 | 28.2 | 42.1 | 71.8 |
| No chronic disease present | 67 | 47.6 (28.1) | 46.7 | 10.7 | 101.3 | 35.8 | 56.7 | 82.1 |
| Chronic disease present | 315 | 54.3 (32.1) | 53.2 | 10.7 | 106.8 | 28.9 | 47.0 | 72.4 |
| Sarcopenia | 90 | 52.5 (20.0) | 51.3 | 12.0 | 98.6 | 28.9 | 47.8 | 76.7 |
| Diabetes mellitus type 2 | 88 | 51.5 (29.1) | 46.8 | 10.2 | 111.8 | 38.6 | 55.6 | 71.5 |
| Kidney disease | 54 | 55.3 (29.8) | 50.5 | 14.2 | 103.1 | 24.1 | 50.0 | 72.2 |
| Osteoporosis | 47 | 66.4 (24.5) | 67.9 | 26.2 | 104.3 | 8.5 | 23.4 | 66.0 |
| Heart failure | 40 | 59.1 (37.9) | 51.8 | 10.8 | 123.8 | 30.0 | 47.5 | 60.0 |
| Thyroid disease | 32 | 57.5 (27.9) | 59.8 | 13.2 | 102.3 | 21.9 | 34.4 | 68.8 |
| Hypertension | 245 | 55.9 (33.0) | 53.7 | 11.0 | 106.8 | 27.8 | 44.9 | 70.2 |
| Hypercholesterolemia | 68 | 54.9 (32.4) | 53.3 | 10.2 | 101.1 | 30.9 | 44.1 | 64.7 |

TUG – timed up and go test; high fat mass %: > 42% for females, > 30% for males; IPAQ – International Physical Activity Questionnaire
