# Supplementary material for: Micronutrient status in nursing home residents: associations with dietary supplementation and health characteristics in the cross-sectional multicentre Nutricare study
Source: Age Ageing. 2025 Oct 9;54(10):afaf290. doi: 10.1093/ageing/afaf290 (PMC12510403; doi:10.1093/ageing/afaf290)
Supplement: Supplementary_table_4_afaf290 [file supplementary_table_4_afaf290.docx]

**Micronutrient status in nursing home residents: associations with dietary supplementation and health characteristics in the cross-sectional multicentre Nutricare study**

Supplementary table 4: Multivariate ordinal logistic regression analysis of the association between serum 25-hydroxyvitamin D (25(OH)D) and haemoglobin levels in relation to different predictors in individuals not supplementing vitamin D (*n* = 166) and iron (*n* = 375)

| Variables | Multivariate ordinal logistic regression analysis | | | | | | Multivariate ordinal logistic regression analysis | | | | | |
| --- | --- | --- | --- | --- | --- | --- | --- | --- | --- | --- | --- | --- |
|  | Dependent variable: three ordered categories of 25(OH)D status | | | | | | Dependent variable: three ordered categories of haemoglobin status | | | | | |
|  | B | SE | *p* | OR | 95% CI | | B | SE | *p* | OR | 95% CI | |
| Summer season (ref.: winter) | 1.00 | 0.33 | 0.003 | 2.72 | 1.41 | 5.22 |  |  |  |  |  |  |
| Moderate IPAQ (ref.: low) | 0.93 | 0.35 | 0.008 | 2.53 | 1.27 | 5.05 |  |  |  |  |  |  |
| High IPAQ (ref.: low) | 1.82 | 0.70 | 0.010 | 6.20 | 1.55 | 24.68 |  |  |  |  |  |  |
| No. of chronic disease | -0.25 | 0.14 | 0.074 | 0.78 | 0.59 | 1.02 | -0.15 | 0.09 | 0.079 | 0.86 | 0.71 | 1.01 |
| Normal hand grip (ref: weak) |  |  |  |  |  |  | 0.96 | 0.24 | < 0.001 | 2.61 | 1.64 | 4.14 |
| Male sex (ref.: female) |  |  |  |  |  |  | 0.75 | 0.24 | < 0.001 | 2.13 | 1.33 | 3.40 |
| CRP |  |  |  |  |  |  | -0.03 | 0.13 | 0.019 | 0.97 | 0.95 | 0.99 |
| Fat mass (%) (ref: low) |  |  |  |  |  |  | 0.83 | 0.38 | 0.031 | 2.29 | 1.08 | 4.88 |
| Number of observations | 167 | | | | | | 310 | | | | | |
| -2 log L | 148.05 | | | | | | 280.88 | | | | | |

Note: OR – odds ratio; IPAQ – International Physical Activity score; CRP – C-reactive protein
